# WHAT'S NEW IN URTICARIA?

**DOI:** 10.4103/0019-5154.55642

**Published:** 2009

**Authors:** Sanjay Ghosh

**Affiliations:** *From the Urticaria Clinic, Institute of Allergic and Immunological Skin Diseases, Kolkata, India.*

**Keywords:** *Urticaia*, *angiedema*, *quality of life*, *urticaria multiforme*, *omalizumab*, *NB UVB*

## Abstract

Urticaria, a perplexing disease of ever-changing explanations, is being renovated almost everyday by newer facts and findings accumulated from different parts of the globe. Cost of the urticaria treatment gradually grows higher and higher whereas the ailment disturbs the quality of life very adversely. Disorder of coagulation cascade has recently thrown some new light into its mechanism. Non-allergic angioedema induced by bradykinin caused by genetic defects and ACE-inhibitors has also been noted. Role of *H. pylori* in the pathogenesis of urticaria has also been re-reviewed. Urticaria could sometimes mimic erythema multiforme and is termed urticaria multiforme. Skin biopsy showed features of vasculitis in good number of urticaria irrespective of clinical features. Contact sensitization showed positive results in certain cases thus proving contact urticaria. Topical clobetasol, systemic omalizumab and NB UVB have shown promising results in certain forms of urticaria.

## Introduction

Urticaria was first described by Hippocrates.[1] Since then it has continued to remain as a perplexing disease, in spite of the enormous advances made in different facets of this disease over the decades. Ever-changing explanations on its pathogenesis have often induced unseen confusions as well as thrilling challenges. Newer frontiers of therapeutic approaches arising regularly on the horizon have always attracted dermatologists.

## What's New?

### Cost and quality of life factors

A study[2] in the Johns Hopkins University, Allergy and Dermatology Ambulatory Clinics showed that for patients with chronic idiopathic urticaria (CIU) the average cost is $2047 annually. Because CIU is primarily an outpatient disease, medication costs alone accounted for 62.5% ($1280) of the total annual cost. Other direct cost includes laboratory, outpatient visit, and emergency department, and hospital visit costs. Indirect costs, including earnings lost owing to travel to outpatient visits and absences from work owing to CIU-related illness, accounted for 15.7% ($322) of the total costs. High medication costs, followed by total indirect costs, result in the largest economic burden for patients with CIU. High medication costs may keep low-income patients at risk for suboptimal treatment and increased burden due to a poorly controlled disease. Total health care costs of chronic urticaria (CU) are comparable to those of other skin diseases like vitiligo and bullous disease.

A national database of hospitalizations in the United States was queried for hospitalizations with a principal diagnosis of angioedema and other major acute allergic disorders (anaphylaxis, urticaria, and allergy unspecified). The angioedema hospitalization rate was 3.3 in 100,000, in 1998, and rose to 4.0 in 100,000, in 2005. In contrast, the combined hospitalization rate for non-angioedema allergic disorders showed an overall decline, and was exceeded by angioedema hospitalization rates after 2000. 24% of the hospitalizations for angioedema were accounted for by adverse effects due to cardiovascular or antihypertensive agents.[3]

CIU is associated with severely impaired quality of life (QoL). Physical health and psychological health were found to be the areas of the QoL most affected in CIU patients. CIU patients frequently suffer from depression and anxiety. The severity of these parameters was found to be positively correlated with the extent of the decrease in QoL.[4]

### Search for the causes

Studies carried out on chronic urticaria (CU) during the last two decades have depicted an autoimmune pathogenesis mediated by functionally active autoantibodies to the high affinity IgE receptor (FcɛRI) or to IgE, which are able to induce histamine release from basophils and mast cells. However, such a mechanism can explain the disease in less than 50% of the patients only. Recent findings[5] have shown that an additional pathogenic mechanism in chronic urticaria patients is the activation of the coagulation cascade, which results in thrombin production. Thrombin is a serine protease that may play a key role in urticaria, by inducing edema through an increase in vascular permeability, mast cell activation and degranulation, and production of the anaphylatoxin C5a. Such a mechanism seems to be active in a majority of CU patients; however, their relationship with anti-FcεRI or anti-IgE autoantibodies is still a matter for research.

Many cases of angioedema are nonallergic reactions, for example, bradykinin-induced angioedema caused by genetic defects and angiotensin-converting enzyme (ACE) inhibitors. This difference is crucial for a successful therapy, particularly when complete emergency care is not available. Five important forms of nonallergic angioedema can be distinguished: hereditary (HAE), acquired (AAE), renin-angiotensin-aldosterone system (RAAS)-blocker-induced (RAE), pseudoallergic angioedema (PAE), and idiopathic angioedema (IAE). A vast majority of nonallergic angioedema are RAE, particularly those caused by ACE inhibitors. The current pharmacotherapy of nonallergic angioedema is not satisfactory. Of late, several new drugs have been developed: A recombinant C1-INH, a kallikrein inhibitor (ecallantide), and a specific bradykinin-B2-receptor antagonist (icatibant), which may improve the treatment of kinin-induced angioedema.[6]

The underlying pathophysiology of urticaria is reported to be mast cell activation, with release of mast cell mediators, predominantly histamine. Substance P is a neuropeptide and has the capacity to provoke histamine release from skin mast cells. Angiotensin-converting enzyme (ACE), widely expressed in skin, is one of the major peptidase for the degradation of substance P. An insertion/deletion polymorphism (I/D) in the ACE gene has been reported to be related to the levels of enzyme. A statistically significant association has not been found between the ACE I/D polymorphism and chronic ordinary urticaria. Further analyses of chronic ordinary urticaria patients have shown that the ACE I/D polymorphism is not associated with the autologous serum skin test status of the patients. However, the frequency of II genotype and I allele are statistically, significantly higher in chronic ordinary urticaria patients with accompanying angioedema, as compared to angioedema-negative patients. Thus, this study[7] shows no evidence of an association between the ACE I/D polymorphism and risk of developing chronic ordinary urticaria. However, it can be a contributing factor to the susceptibility of angioedema in chronic ordinary urticaria.

An Israeli study[8] has shown the effects of *Helicobacter pylori* eradication on chronic urticaria patients, with and without the positive autologous serum skin test (ASST). Eradication of *H. pylori* infection by triple therapy significantly and equally reduces the urticaria activity score (UAS) in CU patients with positive and negative ASST. Another Indian study[9] supported the above findings by demonstrating that the response of the *H. pylori* eradication therapy in infected patients of CIU is significant. It recommended that *H. pylori* should be included in the diagnostic workup of patients with CIU.

### Appearance

Acute annular urticaria is a common and benign cutaneous hypersensitivity reaction seen in children, which manifests with characteristic annular, arcuate, and polycyclic urticarial lesions in association with acral edema. It is mistaken most often for erythema multiforme and occasionally, for a serum-sickness-like reaction. Although these three entities may present in a similar manner, specific clinical features help to distinguish them, and it is important for the clinician to be able to differentiate among them. Because of the frequency of its clinical confusion with erythema multiforme, the term “urticaria multiforme” has been proposed as a more apt description, to highlight the distinctive clinical features of this urticaria variant.[10]

Urticarial vasculitis (UV), an uncommon type of chronic urticaria (CU), exhibits histopathologically leucocytoclastic vasculitis and clinically painful and long lasting (>24 hours) wheals associated with *purpura* or bruising. It is often responsive to oral corticosteroids and poorly to oral antihistamines. Hypocomplementemia and systemic involvement are also commonly reported. An Italian study[11] has recently challenged the diagnostic criteria UV. Biopsies were taken from 312 subjects, with CU unresponsive to oral antihistamines; of these, 47 were histologically diagnosed with UV. Biopsies were taken irrespective of the clinical features of the weal eruption. Other diseases known to be associated with small-vessel vasculitis had previously been excluded. Individual wheals lasted for less than 24 hours in 57.4% of the patients, and pain or tenderness was reported by only 8.6%. Extracutaneous features were present in 81%, hypocomplementemia in 11%, and abnormalities of other laboratory parameters (i.e., raised ESR, microscopic hematuria) in 76.6%. Hydroxyzine was effective in only one patient. Both oral corticosteroids and cinnarizine were effective in a high percentage of patients. Thus, the histopathological diagnostic approach allowed them to identify a large group (47 patients) with UV. Most did not present the clinical (prolonged duration of wheals and bruising) and laboratory features that have previously been described as characteristic of UV.

### Proving the cause

Contact allergy plays a role in chronic urticaria. In a study, out of 121 patients with chronic urticaria, 50 (41%) tested positive to contact allergens. In all patients, avoidance measures led to a complete remission within one month. The authors[12] suggested that testing for contact sensitization could be helpful in the management of chronic urticaria.

### Countdown

Clobetasol propionate 0.05% in a novel foam formulation is safe and effective in the short-term treatment of patients with delayed pressure urticaria, as seen in a randomized, double-blind, placebo-controlled trial.[13]

Three patients with CU refractory to conventional treatment responded to omalizumab therapy.[14] They had therapeutically failed previously with antihistamines, antileukotrienes, and histamine 2 blockers. Systemic steroids provided only temporary relief. Laboratory workup revealed one patient with a low IgE level and elevated anti-IgE receptor antibody level, one patient with an elevated IgE level, but a normal anti-IgE receptor antibody level, and one patient with a very elevated IgE level and an elevated anti-IgE receptor antibody level. All three patients were prescribed omalizumab therapy every two weeks. Two patients had total clearing of urticaria within one week and one patient within six weeks of starting omalizumab therapy. The patient with the elevated anti-IgE receptor antibody level had normalization of the level after starting treatment. Further studies are needed to confirm this effect and better elucidate the mechanism for the observed improvement.

Data regarding narrowband ultraviolet B (NB-UVB) phototherapy in patients with chronic urticaria is limited. An open, controlled study in Turkey[15] was performed to determine whether NB-UVB is effective in treating urticaria in combination with antihistamines. A total of 81 patients with chronic urticaria were recruited, 48 of whom were randomized into the NB-UVB plus antihistamine group. The control group (n = 33) received only antihistamine. Patients were assessed using the urticaria activity score and a visual analog score (VAS). When comparing the groups, the mean urticaria activity score was significantly lower in the NB-UVB group at session 10 (22.6 vs. 27.3) and session 20 (17.4 vs. 20.7). Statistically significant differences were also noted in VAS between the two groups (*P* < 0.01) at three months post treatment. The study concluded that NB-UVB may be an effective complementary treatment for patients with chronic urticaria.
